# Impact of Lean on patient cycle and waiting times at a rural district hospital in KwaZulu-Natal

**DOI:** 10.4102/phcfm.v8i1.1084

**Published:** 2016-07-26

**Authors:** Logandran Naidoo, Ozayr H. Mahomed

**Affiliations:** 1 KwaZulu-Natal Department of Health, Grey’s Hospital, South Africa; 2 Discipline of Public Health Medicine, University of KwaZulu-Natal, South Africa

## Abstract

**Background:**

Prolonged waiting time is a source of patient dissatisfaction with health care and is negatively associated with patient satisfaction. Prolonged waiting times in many district hospitals result in many dissatisfied patients, overworked and frustrated staff, and poor quality of care because of the perceived increased workload.

**Aim:**

The aim of the study was to determine the impact of Lean principles techniques, and tools on the operational efficiency in the outpatient department (OPD) of a rural district hospital.

**Setting:**

The study was conducted at the Catherine Booth Hospital (CBH) – a rural district hospital in KwaZulu-Natal, South Africa.

**Methods:**

This was an action research study with pre-, intermediate-, and post-implementation assessments. Cycle and waiting times were measured by direct observation on two occasions *before*, approximately two-weekly *during*, and on two occasions *after* Lean implementation. A standardised data collection tool was completed by the researcher at each of the six key service nodes in the OPD to capture the waiting times and cycle times.

**Results:**

All six service nodes showed a reduction in cycle times and waiting times between the baseline assessment and post-Lean implementation measurement. Significant reduction was achieved in cycle times (27%; *p* < 0.05) and waiting times (from 11.93 to 10 min; *p* = 0.03) at the Investigations node. Although the target reduction was not achieved for the Consulting Room node, there was a significant reduction in waiting times from 80.95 to 74.43 min, (*p* < 0.001). The average efficiency increased from 16.35% (baseline) to 20.13% (post-intervention).

**Conclusion:**

The application of Lean principles, tools and techniques provides hospital managers with an evidence-based management approach to resolving problems and improving quality indicators.

## Introduction

The White Paper for the Transformation of the Health System in South Africa^1^ set the strategic direction of the district health system (DHS) as the vehicle for primary health care (PHC) services in the democratic South Africa. The district hospital plays an integral role in the DHS by serving as the first point of referral for the PHC clinics; providing supervision, training, and outreach services to the referring facilities;^2^ and acting as a gatekeeper to the higher level of services.^2^

There are numerous challenges that are inherent in the health system that affect district hospitals. Key amongst these challenges that affect the district hospitals are: the quadruple burden of disease,^3^ new and emerging epidemics such as multidrug-resistant (MDR) and extensively drug-resistant (XDR) tuberculosis, poor quality of care, operational inefficiencies, inadequate and inappropriately trained healthcare personnel, inequitable distribution of healthcare personnel, inefficient and inequitable resource allocation (financial and equipment), and deficiencies in managerial capacity and leadership.^4^

The National Service Delivery Agreement (NSDA) has proposed strengthening patient care and satisfaction, accreditation of health facilities for compliance, and improved health infrastructure availability, amongst others, as key to strengthening health systems’ effectiveness.^5^ In order to strengthen patient care and satisfaction and provide patient-centred care, the National Department of Health (NDoH) identified the six most critical areas for patient-centred care^6^ based on the Constitution of South Africa, the Batho Pele principles, the Patients’ Rights Charter, and the National Core Standards (NCS).^7^ The six priority areas focus on three domains namely, Patients’ Rights, Patient Safety, Clinical Governance and Care, and Clinical Support Services. Reducing waiting times and queues (for administration, assessment, diagnosis, pharmacy, surgery, and referral and transfer time), have been identified as a key priority intervention within the Patients’ Rights domain. Prolonged waiting time is a source of patient dissatisfaction with health care^8^ and is negatively associated with patient satisfaction.^9^ Prolonged waiting times in many district hospitals result in many dissatisfied patients, overworked and frustrated staff, and poor quality of care because of the perceived increased workload.

Lean thinking has been introduced as a quality improvement methodology at many hospitals worldwide^10^ to reduce waste or non-value-added elements from the processes so that patients are given greater value.^11^ Waste in a hospital setting is the time spent waiting between and for services. When waste is removed, patients flow smoothly and continuously and this can increase the efficiency, quality, and safety of patient care.^12^

Lean thinking has been applied in the emergency departments (EDs) in many Western countries.^13^ In South Africa, Lean projects have been implemented in many facilities to improve patient flow between their casualty department and other wards, and a tertiary hospital in the Western Cape used Lean principles to reduce the waiting time at the outpatient pharmacy.^14^ A case study conducted using Lean thinking at the orthopaedic outpatient clinic at a secondary-level hospital resulted in a 31% reduction in total time spent at the clinic and a 39% reduction in patient waiting times.^15^

There is a paucity of evidence that Lean (systems or thinking) principles have been used to address operational inefficiencies at district hospitals. We present the findings of our case study in which Lean thinking was applied at the outpatient department (OPD) of a rural district hospital. The aim of the study was to determine the impact of Lean thinking on patient waiting times at the OPD.

## Research methods and study design

### Study design and setting

**FIGURE 1:**
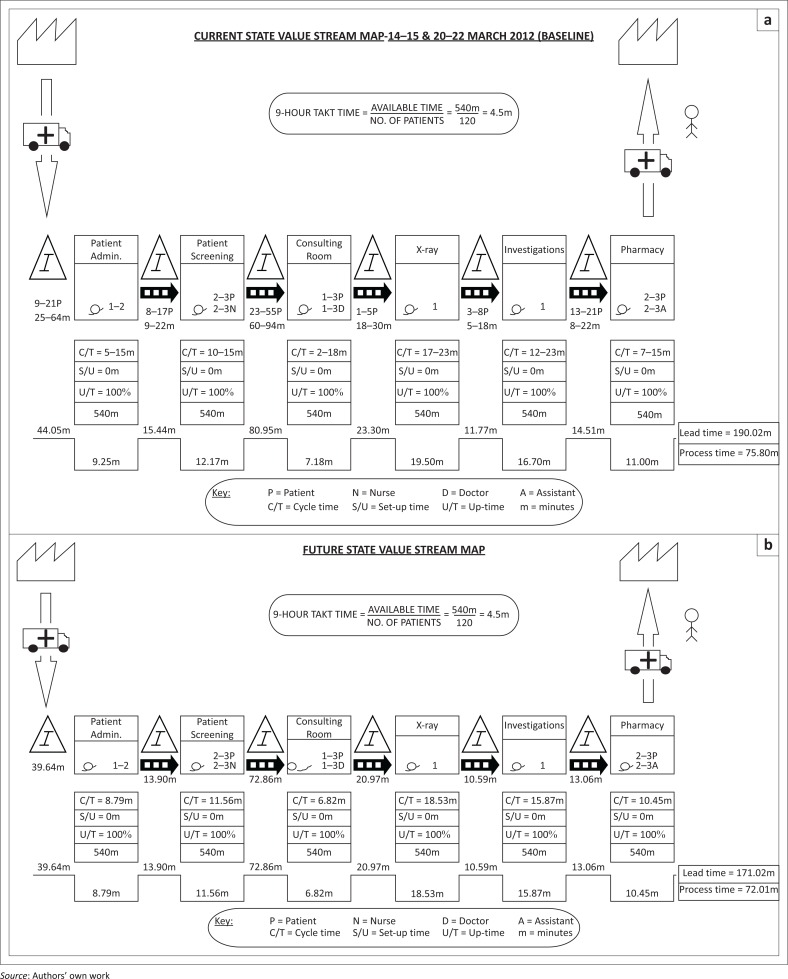
Current and future value stream mapping for outpatient department.

This was an action research study (Figure 1). The study was conducted at the Catherine Booth Hospital (CBH), a 170-bed rural district hospital situated in Amatikulu, in the uThungulu Health District on KwaZulu-Natal’s North Coast. The hospital offers generalist medical and surgical services for both inpatients and outpatients, serving a population of over 200 000 people. uThungulu Health District, as most rural areas in KwaZulu-Natal, mirrors the quadruple burden of disease afflicting South Africa, namely communicable diseases such as HIV and TB, maternal and child morbidity and mortality, non-communicable diseases, and accidental and non-accidental trauma and injuries.

At the time of the study, the OPD consisted of three consulting rooms, a nursing assessment station, a four-bed emergency cubicle, and a waiting area. The study includes all major areas (patient administration, screening, consultation rooms, investigations, X-ray department and/or pharmacy) through which a patient usually has to pass in the health care service delivery process before exiting the hospital.

#### The process and application of Lean tools and techniques

##### Kaizen team meetings^16,17^ (quality improvement team meetings)

After a pre-intervention briefing meeting, two action research cycles were completed and three kaizen team meetings were held.^16,17^ During each meeting, the facilitator delivered a slide presentation that included the latest results of the cycle- and waiting time measurements. The 5-why analysis^17^ and the A3 tool^18^ were used for problem solving.

The pre-intervention briefing meeting sought to explain the process of Lean to *kaizen* team members, and was well understood even though it was a new concept to them. The tools and techniques that were used were simple enough for the *kaizen* team members to use themselves in the meetings. The researcher, however, developed the value stream maps (VSMs) with the results of the cycle and waiting time measurements. This graphical representation of the flow of patients through the OPD gave everyone a more vivid and clear description of the process (Figure 1).

Team members also found the 5-why technique and 5-S tool^17^ valuable in identifying wastes and creating an environment suitable for process enhancement. The targets that were set by the team were realistic. Owing to factors such as establishing patient rapport, counselling patients, standard history-taking and examination, which lengthen the time of the patient consultation, it was impossible to set a greater cycle time reduction target.

##### The A3 reports

Three A3 reports, each of which was developed at every *kaizen* team meeting, proved to be successful as tools with which to engage with *kaizen* team members in problem solving, using techniques such as 5-why. Non-value-adding items (muda), which contributed to long cycle and waiting times, were identified and listed in the A3 tool. An action plan with specific actions, responsible persons, and time frames, was compiled. This was implemented immediately after the *kaizen* team meeting, which then heralded the start of the next action research cycle.

##### Value stream maps

An average outpatient would go through the six service nodes in the sequence described above, each of which is generally preceded by waiting in a queue. It is perceived that a patient values receiving (quality) services at some or all of the service nodes in the OPD rather than waiting in queues (muda). The time spent within each of the service nodes (being ‘processed’) is reflected in the VSM as cycle times, interspersed with the non-value-adding waiting time before each service node.

##### Takt time

The *takt*-based waiting times indicate the total time spent waiting, based on demand for service. It is calculated by the formula: *(takt time)* × *(number of patients waiting)*. The *takt*-based waiting time (*A*) added to the cycle time (*B*) for each service node provides us with the total time (*A+B*) spent by the patient in the OPD, based on demand. Thus the value-adding cycle time (*B*) as a percentage of the total time (*A+B*) reflects the efficiency of the OPD.

The duration of all six service nodes that were open and operational during an ordinary weekday was 540 min (9 h) with 100% uptime (fully functional). The average patient throughput was 120 patients. Therefore, the 9-h *takt* time for the OPD (the cycle time necessary in the process to meet the demand of the patients) was calculated to be 4.5 min. In other words, in an ideal ‘production factory’ setting, the OPD staff would spend 4.5 minutes ‘processing’ each ‘inventory unit’ (patient) in order to finish a 9-h day’s work of 120 units (patients).

##### Wastes and bottlenecks

The problems and wastes that were identified were multifactorial. Patients with chronic medical conditions (such as hypertension and diabetes) presented on random days because there was no booking system, resulting in unpredictable demand. There was no patient-triage system in the OPD. The wastes and bottlenecks included: unnecessary and disorderly movement of staff (and patients), duplication of stationery and work, shortage of equipment, and improper processes and controls. Nurses were also not appropriately triaging patients and conducting appropriate vital signs observations, resulting in back-and-forth movement of patients in OPD, because doctors would not have the information they required during consultations.

#### Measures implemented from kaizen team meetings

A pre-consultation screening tool and a modified patient-triage system, consisting of a list of standing orders for various medical conditions, were implemented for nurses in the Patient Screening node. Patient queues and flows were reorganised such that the movement was more orderly and minimal. A one-way entrance and exit was created in the emergency room to facilitate flow of patients and staff.

**FIGURE 2:**
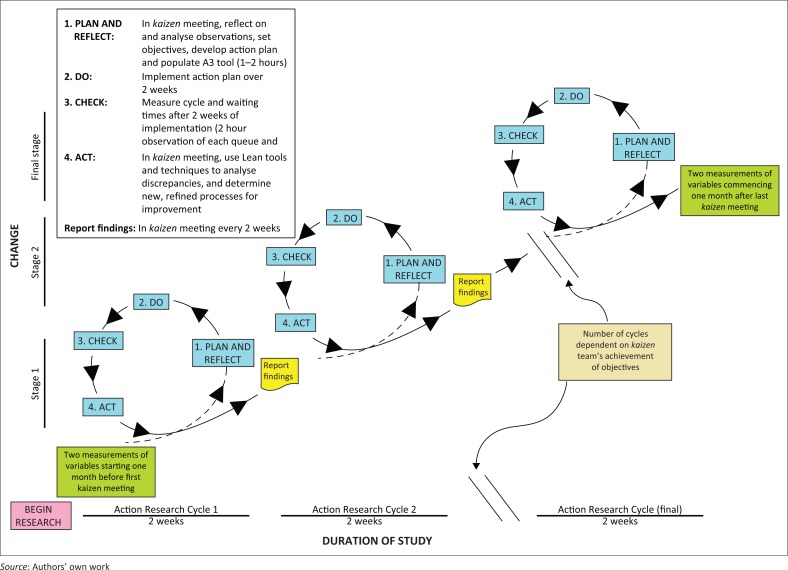
Iterative Plan-Do-Check-Act (PDCA) cycles for continuous quality improvement (19) during the application of Lean in a rural hospital.

A call bell was purchased and installed at the Patient Administration service node so that patients could alert staff that they need to be attended to immediately upon presentation. Lunch and tea breaks were staggered amongst doctors and nurses so as to maintain the 100% uptime. A ‘follow-up’ slip was implemented. Once these were issued to patients who required follow-up, slips would be presented to the nurse in the Patient Screening node on their follow-up visit. This would then expedite service delivery: it would make the nurse aware of the specific reason for the patient’s return, for example, for blood results. A dedicated file containing stationery used only by doctors was created for each Consulting room so as to reduce time searching for such stationery (Figure 2).

#### Study population and sampling

All outpatients observed in a queue preceding each service node during a midweek 2-hour standard observation period (same time, same place, and same days) were included in the sample (pre- and post-Lean implementation). Six service nodes were identified as fundamental in the delivery of outpatient services: Patient administration; Patient screening; Consulting room; X-ray department; Investigations; and Pharmacy.

The number of cycle time measurements (*n*) for each service node was calculated using a formula^19^ consisting of *z* = 1.96 (number of standard deviations from the mean reflecting level of statistical significance); *s* = sample standard deviation of cycle time from the pilot study; *e* = 2 min (absolute amount of acceptable error); and level of confidence = 95%, after measuring five cycle times in a pilot study (Table 1)

**TABLE 1:** Sample size requirements for cycle time measurement at each service node.

Service node	Maximum time	Minimum time	Standard deviation	*n*
Patient administration	13	5	3.54	12
Patient screening	12	8	1.67	3
Consulting room	20	5	5.94	33
X-ray	22	18	1.48	2
Investigations	13	8	2.17	5
Pharmacy	20	12	3.08	9

*Source*: Authors’ own work. Calculated from the pilot study.

Because the number of waiting time measurements (*n*) before each service node during the 2-h observation periods depended on the nature of patient illness and the type of work carried out at the node, *n* was variable during each action research cycle (Table 2).

**TABLE 2:** Sample sizes for waiting times.

Service node	Sample size for waiting times (*n*) during each action research cycle					

	First baseline	Second baseline	Cycle 1	Cycle 2	First post-Lean	Second post-Lean
Patient administration	13	15	10	12	13	13
Patient screening	14	12	14	13	12	14
Consulting room	41	44	45	44	39	45
X-ray	4	5	3	3	4	3
Investigations	8	6	10	12	9	6
Pharmacy	18	14	22	19	18	16

*Source*: Authors’ own work

#### Data sources and data collection

**FIGURE 3:**
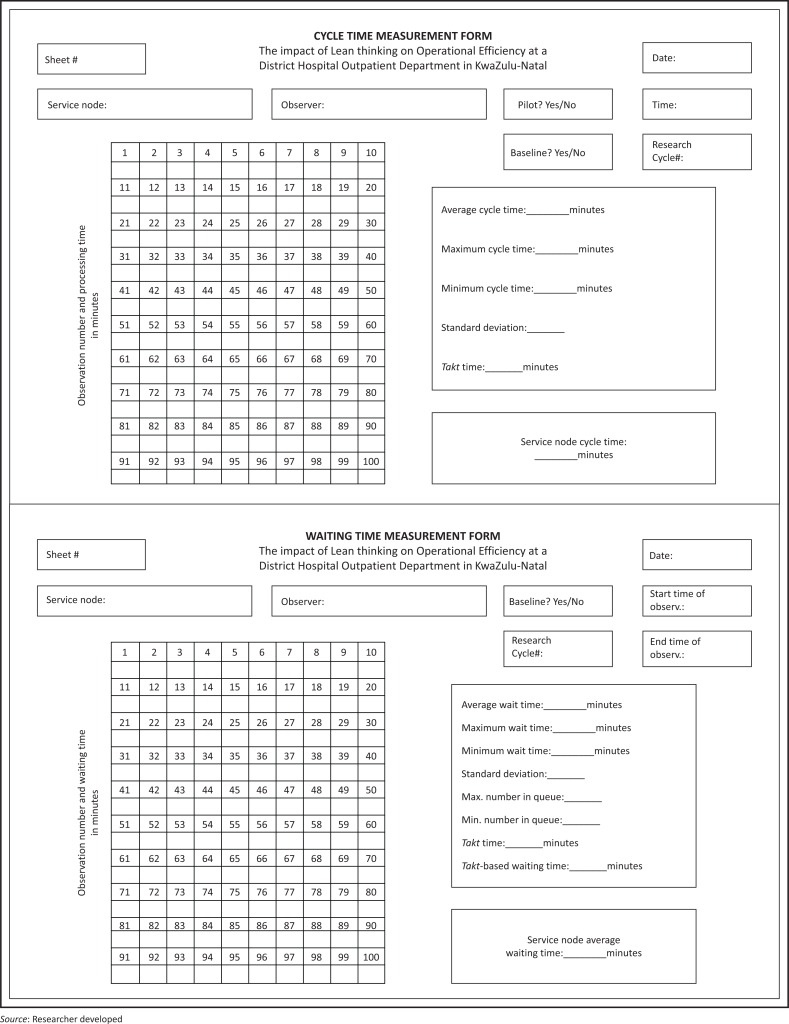
Data collection tools used for measuring cycle and waiting times.

Cycle and waiting times were measured by direct observation on two occasions *before*, approximately two-weekly *during*, and on two occasions *after* Lean implementation. A data collection tool was completed by the professional nurses at each of the six key service nodes in the OPD. The watches of the professional nurses were synchronised at the commencement of the study (Figure 3).

#### Data analysis

The mean cycle and waiting time was calculated for each service point across all measurements. Because pre- and post-intervention groups did not contain paired data (different participants at each pre- and post-intervention time point), they were considered as independent. Therefore, independent samples’ *t*-tests were carried out to compare pre- and post-intervention cycle and waiting times where the assumptions were met. Where the assumptions underlying the *t*-test were not met, the Wilcoxon rank-sum test was used to compare the baseline and post-intervention measurements. The test for trend was done by fitting a linear regression and evaluating the slope of the time variable. Statistical analyses were carried out using the SPSS^®^ software package.

#### Ethical considerations

The study obtained ethics approval from the University of KwaZulu-Natal Biomedical Research Ethics Committee (BE097/11). Permission to conduct the study was obtained from the Hospital Manager, the Department of Health’s uThungulu District Manager, and the KwaZulu-Natal Department of Health Provincial Health Research and Knowledge Management unit.

## Results

### Pre-intervention (baseline) results

The initial process flow analysis, cycle, and waiting times were conducted 14–15 and 20–22 March 2012. Each patient spent, on average, 189.8 min (range 2–23 min) waiting in queues and 75.8 min (range 5–94 min) in the service nodes being ‘processed’. The lengths of the queues varied between the service points with the maximum queue length being at the consultation room (Table 3).

**TABLE 3:** Baseline cycle and waiting times with minimum and maximum queue lengths and cycle and waiting times.

Service node	Baseline cycle time (min)	Baseline waiting time (min)	Max. queue length	Min. queue length	Max. cycle time (min)	Min. cycle time (min)	Max. waiting time (min)	Min. waiting time (min)
Patient administration	9.25	44.14	21	9	15	5	64	25
Patient screening	12.17	15.27	17	8	15	10	22	9
Consulting room	7.18	80.95	55	23	18	2	94	60
X-ray	19.50	23.33	5	1	23	17	30	18
Investigations	16.70	11.93	8	3	23	12	18	5
Pharmacy	11.00	14.16	21	13	15	7	22	8

**Total**	**75.80**	**189.78**	**-**	**-**	**-**	**-**	**-**	**-**

*Source*: Authors’ own work

### Targets

After two baseline measurements of cycle and waiting times at the specified intervals and the baseline survey, targets were set during the *kaizen* meetings and to develop a future-state VSM. The team set a target of 5% reduction in service time and a 10% reduction in waiting time (Table 4).

**TABLE 4:** Target cycle and waiting times.

Service node	Baseline cycle time (min)	Target (min)	Baseline waiting time (min)	Target (min)
Patient administration	9.25	8.79	44.05	39.64
Patient screening	12.17	11.56	15.44	13.90
Consulting room	7.18	6.82	80.95	72.86
X-ray	19.50	18.53	23.30	20.97
Investigations	16.70	15.87	11.77	10.59
Pharmacy	11.00	10.45	14.51	13.06

**Total**	**75.80**	**72.01**	**190.02**	**171.02**

*Source*: Authors’ own work

### Intermediate and post-Lean results

At the end of each action research cycle, the cycle and waiting times were measured to determine trends and to evaluate implemented decisions for feedback during *kaizen* meetings. Because only two further action research cycles were sufficient to reach the targets that were set at the outset, two sets of observations (16–17 May and 4–5 July 2012) and a final post-intervention measurement was carried out on 8–10 August 2012, respectively.

### Cycle times

All six service nodes showed a reduction in cycle times between the baseline assessment and post-Lean implementation measurement. In comparison with baseline and target cycle times (Table 5), post-intervention observations showed that the set targets were met (and exceeded) in the following service nodes: Patient Administration (8 min; *p* = 0.16); Patient Screening (10.33 min; *p* = 0.28); and Investigations (12.2 min; *p* = 0.04). The only significant cycle time reduction was achieved in the Investigations node (27%; *p* < 0.05).

**TABLE 5:** Progressive cycle time measurements.

Service node	Baseline (min)	Cycle 1 (min)	Cycle 2 (min)	Post-Lean (min)	Target (min)	*p*-value for baseline versus post-Lean (*α*= 0.05)	*p*-value for trend (*α* = 0.05)
Patient administration	9.25	8.17	8.08	8.00	8.79	0.16 †	0.14
Patient screening	12.17	11.67	10.33	10.33	11.56	0.28 ‡	0.08
Consulting room	7.18	6.03	6.85	7.03	6.82	0.82 †	0.98
X-ray	19.50	26.50	20.00	19.00	18.53	1.00 ‡	0.60
Investigations	16.70	12.60	12.40	12.20	15.87	0.04 ‡	0.01
Pharmacy	11.00	9.78	11.22	10.78	10.45	0.79 ‡	0.98

**Total**	**75.80**	**74.75**	**68.88**	**67.34**	**72.01**	**-**	**-**

*Source*: Authors’ own work

† *t*-test was used where assumptions were met; otherwise the Wilcoxon test was used.

‡ Wilcoxon test comparing baseline and post-intervention cycle times.

### Waiting times

Post-intervention waiting times also showed reductions for all six service nodes (Table 6), but the targets were met (and exceeded) for the following three service nodes; Patient Administration (37.77 min; *p* = 0.07), Patient Screening (9.15 min; *p* = 0.25), and Investigations (10 min; *p* = 0.03). There was a significant waiting time reduction (*p* < 0.05) for the Investigations node (from 11.93 to 10 min; *p* = 0.03) and (*p* < 0.001) for the Consulting Room node (from 80.95 to 74.43 min; *p* < 0.0001) although the target reduction was not achieved. Significant trends over time were noted in changes in waiting times for the Patient Administration (*p* < 0.05), Patient Screening (*p* < 0.001), and Consulting Room (*p* < 0.001) service nodes.

**TABLE 6:** Progressive waiting time measurements.

Service node	Baseline (min)	Cycle 1 (min)	Cycle 2 (min)	Post-Lean (min)	Target (min)	*p*-value for baseline versus post-Lean (*α* = 0.05)	*p*-value for trend (*α* = 0.05)
Patient administration	44.14	42.20	37.42	37.77	39.64	0.07 †	0.04
Patient screening	15.27	14.00	9.85	9.15	13.90	0.25 ‡	< 0.0001
Consulting room	80.95	78.38	76.86	74.43	72.86	< 0.01†	< 0.0001
X-ray	23.33	23.33	22.33	22.57	20.97	1.00 ‡	0.71
Investigations	11.93	10.80	10.00	10.00	10.59	0.03 ‡	0.16
Pharmacy	14.16	13.41	11.16	14.03	13.06	0.78 ‡	0.69

**Total**	**190.02**	**182.12**	**167.62**	**167.95**	**171.02**	**-**	**-**

*Source*: Authors’ own work

† *t*-test was used where assumptions were met; otherwise the Wilcoxon test was used.

‡ Wilcoxon test comparing baseline and post-intervention cycle times.

### Changes in *takt*-based waiting times and efficiency

Pre-intervention efficiency in the OPD using *takt*-based calculations ranged from 16.00% (with maximum demand) to 16.69% (with minimum demand). In other words, between 16.00 and 16.69% of a patient’s time in the OPD is spent receiving a service. The rest of the time is non-value-adding as it is spent waiting for a service.

The efficiency measured by the total observed cycle time as a percentage of the total time spent in the OPD, based on demand, changed over time since Lean implementation (Table 7). The average efficiency increased from 16.35% (baseline) to 20.13% (post-Lean). During periods of maximum demand, the efficiency increased from 16.00% (baseline) to 17.20% (post-Lean), and during periods of minimum demand, the efficiency increased from 16.69% (baseline) to 23.05% (post-Lean).

**TABLE 7:** Trend in efficiency in the outpatient department (OPD) over the study period.

Research cycle	Baseline		Cycle 1		Cycle 2		Post-Lean	
				
Efficiency based on	Min. demand	Max. demand	Min. demand	Max. demand	Min. demand	Max. demand	Min. demand	Max. demand
Efficiency	16.69%	16.00%	21.66%	16.98%	22.97%	17.16%	23.05%	17.20%
Average efficiency	16.35%		19.32%		20.07%		20.13%	

*Source*: Authors’ own work

### Discussion

An improvement of total cycle time was noted throughout the Lean application process from 75.8 to 67.34 min. These findings are supported by studies of Lean implementation in 15 EDs in the United States, Australia and Canada which showed patient care usually improved after implementation of Lean, with many EDs reporting decreases in length of stay, waiting times, and proportion of patients leaving the ED without being seen.^20^

The targeted total cycle time was exceeded, but was not met in three service nodes: Consulting Room, X-ray Department, and Pharmacy. The implementation of Lean in these three service nodes did reduce the cycle times from baseline, but the targets could not be met over a short period. Extrapolating the trend indicates that the targets for these three nodes would be met over a longer period of Lean implementation. The most likely explanation for this is the inflexible nature of the tasks carried out and the critical shortage of skilled labour in these nodes, including fluctuating staff levels because of service providers being on leave. Additional factors included: equipment problems (for example, X-ray machine breakdown); patient profiles; and disease acuity levels.

The only significant improvement in cycle times was noted in the Investigations section (*p* < 0.05). The primary reason was the implementation of the pre-consultation screening tool, which the nurses used. This tool empowered nurses to make decisions within their scope of practice and without waiting to be given instructions by doctors.

The modified triage system and the changes in layout and flow of patients in waiting areas, were the most important contributors to waiting time reductions for the Patient Screening and Consulting Room nodes. The changes in waiting-room layout allowed for patients to sit closer to the proceeding service node and move in a one-way direction (flow).^12^ Furthermore, the layout allowed for segregation of high-risk groups (such as coughing adult patients) from susceptible groups (children and the elderly) for infection control purposes, thus contributing to the quality aspect of Lean.

The successful implementation of the Lean health practices at this rural district hospital can be attributed to the following critical success factors as previously noted in the literature.^20^

- There was recognition that a problem existed and that improvements were needed.
- A human-centred approach was adopted whereby the employees were involved and part of the solution generation from the onset.
- The medical manager or implementing agent had knowledge of and skills in Lean and educated the staff on the various principles.
- The hospital management endorsed the quality improvement initiative.
- The medical manager acted as the project champion and facilitator.
- Numerous *kaizen* meetings were convened and the solutions were generated based on local needs and adapted accordingly.
- Regular *kaizen* meetings allowed for review of performance and continuous improvements.

### Study limitations and strengths

Although this study was conducted in an OPD setting in only one hospital, this research documents a number of lessons and experiences that other managers in developing countries may find useful. This research used a pragmatic approach to the Lean intervention, rather than an experiment in an artificial setting. The research therefore retained the complexities of the real world, and this considerably improved the chances of reproducing similar results in other settings.

One of the limitations of the study was the lack of enthusiasm from some of the staff in implementing decisions made by the *kaizen* team. Owing to the short study period, the change in culture of the organisation with Lean implementation was impeded, even though this was not one of the objectives of the study.

A further limitation to the study was the postponement of some of the scheduled *kaizen* meetings owing to other staff engagements and priorities. This caused interference with the intervals for measurement of cycle and waiting times in each action research cycle.

The inherent variability in dealing with patients and the nature of the work in the OPD makes the prerequisite of creating stable and predictable flow, an important limitation to the study^21^

Furthermore, there was no comparison group to identify a causal relationship between the intervention and outcomes. However, the action research team felt confident in ascribing the changes observed to the intervention.

## Conclusion and recommendations

The application of Lean principles, tools and techniques provides hospital managers with an evidence-based management approach to resolving problems and improving quality indicators in key focus areas, such as patient waiting times. With the potential benefits of Lean in other departments and facets of health care in a hospital setting, the *kaizen* team should extend their quality improvement efforts to applying Lean elsewhere in the hospital, such as to the wards, theatre, and pharmacy.
